# QMRPF-UKF Master-Slave Filtering for the Attitude Determination of Micro-Nano Satellites Using Gyro and Magnetometer

**DOI:** 10.3390/s101109935

**Published:** 2010-11-05

**Authors:** Peiling Cui, Huijuan Zhang

**Affiliations:** 1 School of Instrumentation Science and Optoelectronics Engineering, Beijing University of Aeronautics and Astronautics, Beijing 100191, China; 2 School of Instrumentation Science and Optoelectronics Engineering, Beijing University of Aeronautics and Astronautics, Beijing 100191, China; E-Mail: lijhem@163.com

**Keywords:** attitude determination, gyro, magnetometer, QMRPF-UKF, master-slave filtering

## Abstract

In this paper, the problem of estimating the attitude of a micro-nano satellite, obtaining geomagnetic field measurements via a three-axis magnetometer and obtaining angle rate via gyro, is considered. For this application, a QMRPF-UKF master-slave filtering method is proposed, which uses the QMRPF and UKF algorithms to estimate the rotation quaternion and the gyro bias parameters, respectively. The computational complexicity related to the particle filtering technique is eliminated by introducing a multiresolution approach that permits a significant reduction in the number of particles. This renders QMRPF-UKF master-slave filter computationally efficient and enables its implementation with a remarkably small number of particles. Simulation results by using QMRPF-UKF are given, which demonstrate the validity of the QMRPF-UKF nonlinear filter.

## 1. Introduction

Spacecraft attitude is important for attitude control in many space missions. In the last four decades, a great number of research works have been devoted to the problem of estimating spacecraft attitude based on a sequence of noisy vector observations, resolved in the body-fixed coordinate system and in a reference system [1–7].

For the attitude sensor, the sun sensor, Earth sensor, star tracker, three-axis magnetometer, and gyro *etc*, are often used. The attitude estimation problem possesses an undesirable strong nonlinearity. The filtering-based methods that were developed in the 1980s embedded the attitude determination problem in the framework of stochastic filtering. The mostly used filtering method is the extended Kalman filter (EKF) [2,8]. Nevertheless, poor performance or even divergence arising from the linearization implicit in the EKF has led to the development of other filters [3,9,10]. Another often used method is the unscented Kalman filter (UKF), also known as the sigma-point filter [4]. Unscented filters are essentially based on second or higher-order approximations of nonlinear functions, which are used to estimate the mean and covariance of the state vector. In [4], an unconstrained three-component vector is used, based on the generalized Rodrigues parameters to represent an attitude error quaternion. The state vector includes the attitude error and bias vectors. UKF, as a Kalman filter mechanization, is sensitive to the statistical distribution of the stochastic processes driving the dynamic model: non-Gaussian distributions guarantee nonoptimality of the estimates. Recently, a new method using the particle filtering (PF) technique has been proposed for a spacecraft’s attitude estimation [1,5–7]. Based on the concept of sequential importance sampling and the use of Bayesian theory, particle filtering is particularly useful in dealing with nonlinear and non-Gaussian problems. However, the most notorious disadvantage of a particle filter is its formidable computational complexity, since hundreds even thousands of particles are usually needed to achieve required approximation accuracy, and then it is difficult to achieve real time performance. A particle filtering algorithm [1] copes with the curse of computational complexity related to PFs by using an efficient initialization procedure, along with an importance weight cooling schedule and particle set portioning. This method is applicable to gyroless attitude determination setting, and the state vector is composed of a unit norm quaternion and a three-element angular-rate vector. In [5], particle filtering for attitude estimation using a minimal local-error representation (MLERPF) is proposed. The main character of MLERPF is that it does not work well when the number of particles is not large enough, which brings limitation to some applications.

This paper develops a QMRPF-UKF master-slave filter and gives its implementation in the case of a three-axis stabilized micro-nano satellite, obtaining noisy geomagnetic field measurements via a three-axis magnetometer and obtaining the noisy angle rate via gyro. In order to reduce the computational cost rising from the increased state dimensions, the quaternion multiresolution particle filter (QMRPF) algorithm is used as the master filter to estimate the attitude quaternion, and the unscented Kalman filter algorithm is used as the slave filter to determine the gyro bias parameters. On the other hand, the new estimator copes with the curse of computational complexity related to the particle filtering technique by introducing multiresolution approach that permits a significant reduction in the number of particles [11]. This renders the QMRPF-UKF master-slave filter computationally efficient and significant computational savings of QMRPF master filter is achieved by drastically reducing the needed number of particles. The performance of QMRPF-UKF in various scenarios is studied and compared with MLERPF method, which demonstrates the validity of QMRPF-UKF nonlinear filter.

This paper is organized as follows. In Section 2, the system model of the attitude determination of a three-axis stabilized micro-nano satellite by using gyro and magnetometer is given. In Section 3, the QMRPF-UKF master-slave filter is described in detail. In Section 4, the QMRPF-UKF master-slave filter for the attitude determination problem is verified on the basis of its estimation accuracy and computational time. We draw some conclusions in Section 5.

## 2. System Model

### 2.1. Quaternion Process Model

It is known that the body angular motion can be described in terms of the attitude quaternion by the following equation:

(1) $$q_{k+1}=\Omega (\omega_{k})q_{k}$$

where $\omega_{k}={\left[\begin{array}{lll} \omega_{xk} & \omega_{yk} & \omega_{zk} \end{array}\right]}^{T}$ denotes the angular velocity vector of the rotation of *B* with respect to *R*. *q_k_* denotes the quaternion of rotation from a given reference frame *R* onto the body frame *B* at times *k* = 1,2, ..., ∞. The quaternion is constructed from vector part *q̄_k_* and scalar part *q*_4_*_k_*:

(2) $$q_{k}={\left[{\bar{q}}_{k}{}^{T}\;\;\;\;\;\;\;\;q_{4k}\right]}^{T}$$

Assuming that *ω_k_* is constant during the sampling time interval Δ*t*, the orthogonal transition matrix Ω(*ω_k_*) is expressed using *ω_k_* and computed as follows:

(3) $$\Omega (\omega_{k})={\left[\begin{array}{cc} \text{cos}\left(0.5\left\|\omega_{k}\right\|\Delta t\right)I_{3\times 3}-\left[\psi_{k}\times \right] & \psi_{k}\\ -\psi_{k}^{T} & \text{cos}\left(0.5\left\|\omega_{k}\right\|\Delta t\right) \end{array}\right]}_{4\times 4}$$

(4) $$\psi_{k}=\frac{\text{sin}\left(0.5\left\|\omega_{k}\right\|\Delta t\right)\omega_{k}}{\left\|\omega_{k}\right\|}$$

where [*ψ_k_* ×] denotes the cross-product matrix associated with the vector *ψ_k_*.

### 2.2. Rate Sensor Measurement Model

A common sensor that measures the angular rate is a rate-integrating gyro. For this sensor, a widely used model is given by:

(5) $${\tilde{\omega }}_{k}=\omega_{k}+\beta_{k}+\eta_{v,k}$$

(6) $$\beta_{k+1}=\beta_{k}+\eta_{u,k}$$

where *ω̃_k_* is the measured rate; *β_k_* is the gyro bias vector; *η_v,k_* and *η_u,k_* are independent zero-mean Gaussian white-noise processes with:

(7) $$E\left\{\eta_{v}(t)\eta_{v}^{T}(\tau )\right\}=\sigma_{v}^{2}\delta (t-\tau )I_{3\times 3}$$

(8) $$E\left\{\eta_{u}(t)\eta_{u}^{T}(\tau )\right\}=\sigma_{u}^{2}\delta (t-\tau )I_{3\times 3}$$

where *E*{·} denotes expectation and *δ*(*t* – *τ*) is the Dirac_delta function.

### 2.3. Observation Model

A widely used attitude measurement model is given by:

(9) $$b_{k}=A(q_{k})r_{k}+\delta b_{k}$$

where *b_k_* is the body-frame vector and *r_k_* is the reference-frame vector; *δb_k_* denotes the measurement noise process, with known probability density function (PDF) denoted as *δb_k_* ∼ *p_δb_k__*; where *A*(*q_k_*) denotes the rotation matrix that brings the axes of *R* onto the axes of *B* at time *k*. The attitude matrix is related to the quaternion by:

(10) $$A(q_{k})=[{\left(q_{4k}\right)}^{2}-{\bar{q}}_{k}{}^{T}{\bar{q}}_{k}]I_{3\times 3}+2{\bar{q}}_{k}{\bar{q}}_{k}{}^{T}-2q_{4k}[{\bar{q}}_{k}\times ]$$

## 3. QMRPF-UKF Master-Slave Filter

**Figure 1.**
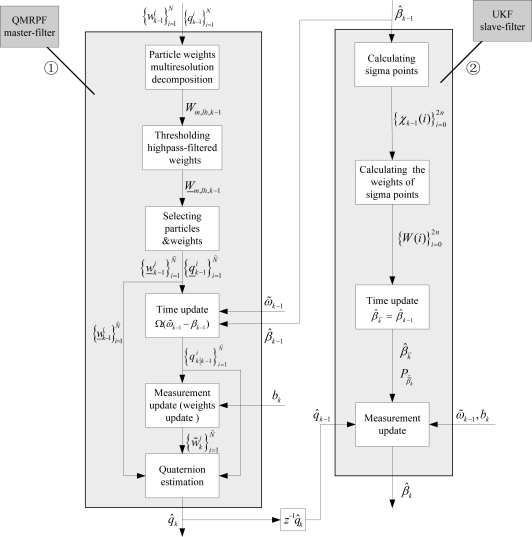
QMRPF-UKF master-slave filter with multiresolution approach embedded at time *k* = *M*.

In this Section, the QMRPF-UKF master-slave filtering method is proposed, which uses the QMRPF and UKF algorithms to estimate the rotation quaternion and the gyro bias parameters, respectively. Section 3.1 formulates a detailed process of QMRPF master-filter. Section 3.2 describes the UKF slave-filter process. The flow chart of the QMRPF-UKF master-slave filter with multiresolution approach embedded at time *k* = *M* is shown in Figure 1, where ① denotes the master filter, and ② denotes the slave filter.

### 3.1. QMRPF Master-Filter

QMRPF master-filter is provided to estimate the quaternion from pairs of vector observations, and computational efficiency of quaternion particle filter (QPF) is achieved by using the spatial-domain multiresolutional approach. This section is divided into three parts: QMRPF master-filter mathematical model, the complete step sum-up of QMRPF master-filter, quaternion particle filter with multiresolution embedded.

#### A. QMRPF Master-Filter Mathematical Model

Taking attitude quaternion as state variable, the mathematical model of QMRPF master-filter is built. State equation is

(11) $$q_{k}=\Omega ({\tilde{\omega }}_{k-1}-\beta_{k-1})q_{k-1}$$

Measurement equation is Equation (9). From Equation (11), it can be seen that, the gyro bias estimation at time *k* – 1 will be used for estimating the attitude quaternion at time *k*.

#### B. Complete Step Sum-up of QMRPF Master-filter

The time is supposed to be *k* = *M* when the multiresolution approach is embedded into the quaternion particle filter.

1. Initializing the quaternion particles ${\left\{q_{0}^{i}\right\}}_{i=1}^{N}$ and setting quaternion weights as $\left\{w_{0}^{i}\right\}=1/N,$ *i* = 1, ..., *N*.
2. For *k* < *M*, quaternion particle filter is used to estimate the attitude quaternion *q̂_k_* at time *k*, and the quaternion particles ${\left\{q_{k}^{i}\right\}}_{i=1}^{N}$ and ${\left\{w_{k}^{i}\right\}}_{i=1}^{N}$ are also obtained.
3. For *k = M*, QMRPF algorithm is adopted to determinate the rotation quaternion *q̂_k_* at time *k*, the number *N* of particles is reduced to be *Ñ*. ${\left\{{\underline{q}}_{k}^{i}\right\}}_{i=1}^{\tilde{N}}$ and ${\left\{{\underline{w}}_{k}^{i}\right\}}_{i=1}^{\tilde{N}}$ are derived at time *M*.
4. For *k* > *M*, the basic step is the same as step 2, but the number of quaternion particles is *Ñ*.

#### C. Quaternion Particle Filter with Multiresolution Embedded

In the following, we will show how *q̂_k_* is estimated when the multiresolution approach is embedded into the quaternion particle filter at time *k* = *M*.

(1) Particle weights multiresolution decomposition

A partition of *N* samples into *N*/2*^L^* blocks is used where *L* is the level number of multiresolution decomposition, and then particle weights of every block are decomposed into lowpassed and highpassed components.

(12) $$W_{m,lh,k-1}=\left[\begin{array}{c} {\tilde{w}}_{m,l_{1},k-1}^{1}\\ {\tilde{w}}_{m,h_{1},k-1}^{1}\\ \vdots \\ {\tilde{w}}_{m,h_{L},k-1}^{1}\\ \vdots \\ {\tilde{w}}_{m,h_{L},k-1}^{2^{L-1}} \end{array}\right]=T\left[\begin{array}{c} {\tilde{w}}_{m,k-1}^{1}\\ \vdots \\ {\tilde{w}}_{m,k-1}^{2^{L}} \end{array}\right],m=1,\cdots ,{N/2}^{L}$$

where *T* denotes an orthogonal wavelet decomposition transform matrix; *m* denotes the *m*-th block; ${\left\{{\tilde{w}}_{m,k-1}^{i}\right\}}_{i=1}^{2^{L}}$ are the particle weights of the *m*-th block at time *k* – 1; ${\tilde{w}}_{m,l_{1},k-1}^{1}$ is the lowpass-filtered particle weight; ${\left\{{\tilde{w}}_{m,h_{j},k-1}^{i}\right\}}_{i=1}^{2^{j-1}}$ are the highpass-filtered particle weights at level *j*(*j* = 1, ..., *L*), and the number of weights at scale *j* is 2*^j^*^−1^.

(2) Thresholding the highpass-filtered particle weights

A simple thresholding is carried out on the highpass-filtered particle weights of every block:

(13) $${\underline{w}}_{m,h_{j},k-1}^{i}=\{\begin{array}{l} {\tilde{w}}_{m,h_{j},k-1}^{i},\;\left|{\tilde{w}}_{m,h_{j},k-1}^{i}\right|>T_{s}\\ 0,\;\;\;\;\;\;\;\;\left|{\tilde{w}}_{m,h_{j},k-1}^{i}\right|\leq T_{s} \end{array}\;\;\;(j=1,\cdots ,L;i=1,\cdots ,2^{j-1})$$

where *T_s_* is the wavelet threshold. Thus we have the thresholded weights:

(14) $${\underline{W}}_{m,lh,k-1}={\left[{\tilde{w}}_{m,l_{1},k-1}^{1},{\underline{w}}_{m,h_{1},k-1}^{1},\cdots ,{\underline{w}}_{m,h_{L},k-1}^{1},\cdots ,{\underline{w}}_{m,h_{L},k-1}^{2^{L-1}}\right]}^{T}$$

(3) Quaternion particles selection

The thresholded weight *W*_*m,lh,k*−1_ corresponds to quaternion particles ${\left\{{\underline{q}}_{m,lh,k-1}^{i}\right\}}_{i=1}^{2^{L}}$. Therefore, we can just propagate the elements in ${\underline{q}}_{m,k-1}^{i}=T^{T}{\underline{q}}_{m,lh,k-1}^{i}$ which corresponds to the distinct elements in *W_m,k_*_–1_ = *T^T^W_m,lh,k_*_–1_. For the repeated elements of *W_m,k_*_–1_, we can propagate one corresponding representative element in ${\underline{q}}_{m,k-1}^{i}$, The number of quaternion particles is selected to be *Ñ* by the thresholding operation, which can significantly reduce the computation in propagation while maintaining performance. Some detailed information about the multiresolution process can be referenced to [11].

Now let ${\left\{{\underline{q}}_{k-1}^{i}\right\}}_{i=1}^{\tilde{N}}$ and ${\left\{{\underline{w}}_{k-1}^{i}\right\}}_{i=1}^{\tilde{N}}$ denote *Ñ* independent quaternion particles and their associated weights, *Ñ* denotes the number of quaternion particles selected.

(4) Time update

The prediction values ${\left\{q_{k|k-1}^{i}\right\}}_{i=1}^{\tilde{N}}$ are obtained from Equation (11), where *β_k_*_–1_ is the estimation of the gyro bias at time *k* – 1 via UKF algorithm.

(5) Measurement update (the update of weight):

(15) $$w_{k}^{i}\propto p_{b_{k}|q_{k}}(b_{k}|q_{k|k-1}^{i}){\underline{w}}_{k-1}^{i}=p_{\delta b_{k}}(b_{k}-A(q_{k|k-1}^{i})r_{k}){\underline{w}}_{k-1}^{i},i=1,...,\tilde{N}$$

where $p_{b_{k}|q_{k}}(b_{k}|q_{k|k-1}^{i})$ is the likelihood probability of the measurement *b_k_* associated to a given quaternion $q_{k|k-1}^{i}$, *p_δb_k__* is the probability density function of *δb_k_*. Particle weights $w_{k}^{i}$ are normalized to ${\tilde{w}}_{k}^{i}$:

(16) w˜ki=wki∑i=1N˜wki,(i=1,...,N˜)

(6) Quaternion estimation

At time *k*, *Ñ* weighted quaternion samples are available. To obtain the filtered quaternion via minimum mean square error (MMSE) approach, the following minimization problem is solved [6]:

(17) $$\underset{{\hat{A}}_{k}}{\text{min}}\sum_{i=1}^{\tilde{N}}{\tilde{w}}_{k}^{i}{\left\|A_{k}(q_{k/k-1}^{i})-{\hat{A}}_{k}\right\|}_{F}^{2},\;\text{subject}\;\text{to}\;{\hat{A}}_{k}{}^{T}{\hat{A}}_{k}=I_{3\times 3}$$

where *Â_k_* denotes the orthogonal attitude matrix associated with the filtered quaternion, and ||·||*_F_* is the Frobenius norm. The constrained minimization problem (17) is equivalent to the unconstrained maximization problem in quadratic form as follows:

(18) $$\underset{{\hat{A}}_{k}}{\text{max}\;\mathrm{tr}}\left[{\hat{A}}_{k}{}^{T}\sum_{i=1}^{\tilde{N}}{\tilde{w}}_{k}^{i}A_{k}(q_{k/k-1}^{i})\right]=\underset{{\hat{q}}_{k}}{\text{max}}\;{\hat{q}}_{k}{}^{T}K{\hat{q}}_{k}$$

with:

(19) $$K=\left[\begin{array}{cc} B_{k}+B_{k}{}^{T}-I_{3\times 3}tr(B_{k}) & z\\ z^{T} & tr(B_{k}) \end{array}\right],z=\left[\begin{array}{c} B_{k}(3,2)-B_{k}(2,3)\\ B_{k}(1,3)-B_{k}(3,1)\\ B_{k}(2,1)-B_{k}(1,2) \end{array}\right]$$,

Letting:

(20) $$B_{k}=\sum_{i=1}^{\tilde{N}}{\tilde{w}}_{k}^{i}A({\hat{q}}_{{}_{k|k-1}}^{i})$$

As in Davenport’s well-known q-method, the quaternion that solves the maximization problem of Equation (18) is the normalized eigenvector corresponding to the largest eigenvalue of *K*:

(21) $$K{\hat{q}}_{k}=\lambda_{\text{max}}{\hat{q}}_{k}$$

Then the attitude quaternion estimation *q̂_k_* at time *k* = *M* is obtained.

### 3.2. UKF Slave-Filter

The gyro output can’t be used for attitude estimation directly for the reason that the gyro has a bias vector, and thus the gyro bias is considered in the estimation process. The UKF slave-filter is designed to estimate the gyro bias vector. Taking gyro bias as state variable, the state space model of slave filter, UKF, is built on the basis of gyro bias model and vector observation model. State equation is Equation (6), and observation equation is:

(22) $$b_{k}=A(\Omega ({\tilde{\omega }}_{k-1}-\beta_{k-1})q_{k-1})r_{k}+\delta b_{k}$$

The noise variance matrix of state model and observation model are supposed to be *Q*_*σ*_*u*_^2^_ and *R_δb_*, respectively. $Q_{\sigma_{u}{}^{2}}=\sigma_{u}^{2}I_{3\times 3}$.

It can be seen that, the state equation is linear and the observation equation is nonlinear. Moreover, the attitude quaternion at time *k* – 1 will be used to estimate the gyro bias at time *k*. The steps of gyro bias estimation using UKF is as follows:

1. Initialization
  (23) $${\hat{\beta }}_{0}=E[\beta_{0}],\;P_{\beta_{0}}=E[(\beta_{0}-{\hat{\beta }}_{0})\;{\left(\beta_{0}-{\hat{\beta }}_{0}\right)}^{T}]$$
  where *β*_0_ and *β̂*_0_ are the real value and estimation value of gyro bias at the starting time, respectively. *P_β_0__* denotes the estimation error variance matrix at the starting time.
2. Calculating sigma points
  (24) $$\left\{ \begin{array}{l} \chi_{k-1}(0)={\hat{\beta }}_{k-1}\\ \chi_{k-1}(i)={\hat{\beta }}_{k-1}+\sqrt{n+\tau }{\left(\sqrt{P_{{\hat{\beta }}_{k-1}}}\right)}_{i}\\ \chi_{k-1}(i+n)={\hat{\beta }}_{k-1}-\sqrt{n+\tau }{\left(\sqrt{P_{{\hat{\beta }}_{k-1}}}\right)}_{i} \end{array}\right.,i=1,\ldots ,n$$
  where *β̂_k_*_–1_ and *P*_*β̂*_*k*–1__ denote the estimation value and estimation error variance matrix at time *k* – 1, respectively; *n* denotes state dimension, and for this application, *n* = 3. Supposing that the estimation error variance matrix *P*_*β̂*_*k*–1__ = *AA^T^*, ${\left(\sqrt{P_{{\hat{\beta }}_{k-1}}}\right)}_{i}$ denotes the *i*-th column of matrix *A*.
3. Calculating the weights of sigma points
  The weights corresponding to the sigma points are given by:
  (25) $$\left\{ \begin{array}{l} W(0)=\tau /(n+\tau )\\ W(i)=1/[2(n+\tau )]\\ W(n+i)=1/[2(n+\tau )] \end{array}\right.,i=1,\ldots ,n$$
4. Time update
  The predicted mean and error variance matrix of the gyro bias at time *k* are given by:
  (26) $${\hat{\beta }}_{\bar{k}}={\hat{\beta }}_{k-1},\;P_{{\hat{\bar{\beta }}}_{k}}=P_{{\hat{\beta }}_{k-1}}+Q_{\sigma_{u}{}^{2}}$$
5. Measurement update
  (27) $${\hat{b}}_{k|k-1}(i)=A(\Omega ({\tilde{\omega }}_{k-1}-\chi_{k-1}(i)){\hat{q}}_{k-1})r_{k},i=0,1,...,2n$$
  The prediction mean of the measurement vector at time *k* is calculated using a weighted sum of the points *b̂_k_*_|_*_k_*_–1_(*i*), which is given by:
  (28) $${\hat{b}}_{\bar{k}}=\sum_{i=0}^{2n}W(i){\hat{b}}_{k|k-1}(i)$$
  The measurement variance and cross correlation between *β̂_k̄_* and *b̂_k̄_* are computed by:
  (29) $$P_{b_{k}b_{k}}=\sum_{i=0}^{2n}W(i)({\hat{b}}_{k|k-1}(i)-{\hat{b}}_{\bar{k}})\;{\left({\hat{b}}_{k|k-1}(i)-{\hat{b}}_{\bar{k}}\right)}^{T}+R_{\delta b}$$
  (30) $$P_{\beta_{k}b_{k}}=\sum_{i=0}^{2n}W(i)(\chi_{k-1}(i)-{\hat{\beta }}_{\bar{k}})\;{\left({\hat{b}}_{k|k-1}(i)-{\hat{b}}_{\bar{k}}\right)}^{T}$$
  The Kalman gain is:
  (31) $$K_{k}=P_{\beta_{k}b_{k}}P_{b_{k}b_{k}}^{-1}$$
  Then the estimation value and estimation error variance matrix of gyro bias at time *k* are described by:
  (32) $${\hat{\beta }}_{k}={\hat{\beta }}_{\bar{k}}+K_{k}(b_{k}-{\hat{b}}_{\bar{k}})$$
  (33) $$P_{{\hat{\beta }}_{k}}=P_{{\hat{\bar{\beta }}}_{k}}-K_{k}P_{b_{k}b_{k}}K_{k}{}^{T}$$

## 4. Simulation Results and Analysis

In our simulation, the micro-nano satellite orbit is in sun synchronization orbit with 900 km altitude. Gyro and magnetometer are integrated for attitude determination. The magnetic field reference is modeled using a 10th-order International Geomagnetic Reference Field model. Magnetometer noise is modeled by a zero-mean Gaussian white-noise process with a standard deviation of 50 nT. The gyros’ output is contaminated with measurement noise having two components: white, zero-mean Gaussian process with intensity ${\left(0.31623\mu \text{rad}/s^{\frac{1}{2}}\right)}^{2}$ and gyro bias modeled as integrated Gaussian white noise with intensity ${\left(3.1623\times 10^{-4}\mu \text{rad}/s^{\frac{3}{2}}\right)}^{2}$. The initial attitude error angle is 0°, the initial gyro bias is 0.2(°/h) on each axis, the gyro bias variance matrix is [0.2(°/h)]^2^ × *I*_3×3_, the measurement noise matrix of magnetometer is 50^2^ *I*_3×3_ (nT)^2^, the sampling frequency is 10 Hz, simulation time is 3,000 s. The initial particle number of QMRPF-UKF method is 600. The threshold is 0.5 × 10^−4^. The starting time of performing multiresolutional transform is the 1,000 s. QMRPF-UKF master-slave filtering is used to perform the attitude determination of this micro-nano satellite. The codes are run on a computer, the processor speed of which is 2.5 GHz and memory is 2 Gbyte. In testing the filter, we look for two filter attributes: computational cost and accuracy.

**Figure 2.**
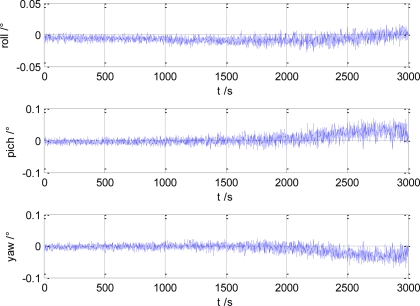
Estimation error of attitude angle.

**Figure 3.**
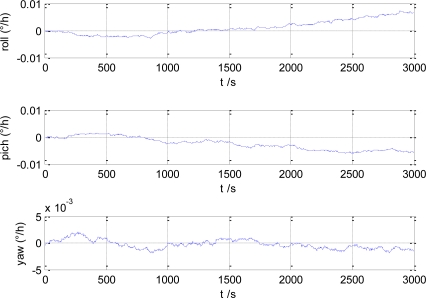
Estimation error of gyro bias.

The estimation error of gyro/magnetometer integrated attitude angle estimation is shown in Figure 2. In Figure 3, the gyro bias estimation error is given. It can be seen that the attitude angle estimation error is smaller than 0.1°, and the gyro bias estimation error is smaller than 0.01°/h.

**Figure 4.**
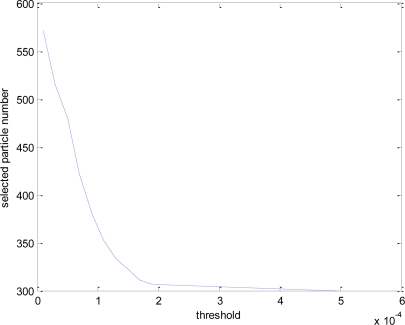
Selected particle number after performing QMRPF-UKF with different thresholds.

Here, we run QMRPF-UKF algorithm in 50 runs of Monte Carlo simulation with different thresholds to validate the effectiveness and complexity of QMRPF-UKF for the attitude determination of a micro-nano satellite. The performance is measured via RMS (root mean square) estimation error of Monte Carlo simulation. The smaller the RMS estimation error resulting from the filter is, the better the filter performance is. Figure 4 gives the selected particles numbers after performing multiresolution particle filter with different thresholds. RMS error and the computational cost of QMRPF-UKF with different thresholds are given in Table 1. It can be seen that, the larger the threshold is, the smaller the number of surviving particles is. With the increase of threshold, the computational time is decreased, and the filtering varies in estimation precision.

QMRPF-UKF is compared with the existing method, MLERPF, in terms of RMS error and computational cost, as shown in Table 1. It can be seen that, QMRPF-UKF outperforms MLERPF significantly in terms of computational cost and QMRPF-UKF is comparable to MLERPF in terms of estimation precision. For MLERPF, the state dimension of performing particle filter is six, which is larger than that of QMRPF-UKF. Moreover, in the calculating process of MLERPF, the particle number is invariant. For QMRPF-UKF master-slave filter, the filtering performance is improved from two aspects. On the one hand, in order to reduce the computational cost rising from the augment of the state dimensions, QMRPF algorithm is used as the master filter to estimate the attitude quaternion, and UKF is used as the slave filter to determine the gyro bias parameters. On the other hand, QMRPF copes with the curse of computational complexity related to the particle filtering technique by introducing multiresolution approach that permits a significant reduction in the number of particles.

Three simulation scenarios are carried out to verify the convergence performance of QMRPF-UKF. The first one is that, the initial attitude error angle is 50°, 50°, and 50° for each axis, respectively. The second one is that, the initial attitude error angle is −70°, 40°, and 100° for each axis, respectively. The third one is that, the initial attitude error angle is −60°, 30°, and 130° for each axis, respectively. These are added into the initial condition of attitude estimation. For each simulation scenario, other parameters are same as the above mentioned ones. QMRPF-UKF convergences in each simulation. The attitude angle estimation error is smaller than 0.1°, and the gyro bias estimation error is smaller than 0.01°/h.

## 5. Conclusions

We have developed a computationally efficient QMRPF-UKF master-slave filtering method, to solve the problem of gyro/magnetometer integrated attitude determination of a three-axis stabilized micro-nano satellite. Computational efficiency of the proposed filtering is achieved by combining the master-filter with slave-filter and by adopting the spatial-domain multiresolutional approach to reduce the needed number of particles while maintaining comparable estimation accuracy. The larger the threshold is, the fewer the number of surviving particles, and the filtering varies in estimation precision.

## Figures and Tables

**Table 1.** Comparison of QMRPF-UKF with MLERPF in terms of RMS error and computational cost.

	**Threshold of QMRPF-UKF**							**MLERPF**
	**1 × 10^−5^**	**5 × 10^−5^**	**7 × 10^−5^**	**1.3 × 10^−4^**	**1.7 × 10^−4^**	**1.9 × 10^−4^**	**5 × 10^−4^**	
**RMS error (°)**	0.0115	0.0137	0.0128	0.0108	0.0134	0.0124	0.0126	0.020
**Computational cost (s)**	223	221	219	194	180	171	160	1840
